# Glucose-6-Phosphate Upregulates Txnip Expression by Interacting With MondoA

**DOI:** 10.3389/fmolb.2019.00147

**Published:** 2020-01-09

**Authors:** Xueyun Zhang, Tao Fu, Qian He, Xiang Gao, Yan Luo

**Affiliations:** ^1^Department of Biochemistry, School of Medicine, Cancer Institute of the Second Affiliated Hospital, Zhejiang University, Hangzhou, China; ^2^Key Laboratory of Cancer Prevention and Intervention of China National Ministry of Education, Hangzhou, China

**Keywords:** Txnip, glucose metabolism, MondoA/Mlx, G6P, molecule recognition

## Abstract

The major metabolic fates of glucose in cells are glycolysis and the pentose phosphate pathway, and they share the first step: converting glucose to glucose-6-phosphate (G6P). Here, we show that G6P can be sensed by the transcription factor MondoA/Mlx to modulate Txnip expression. Endogenous knockdown and EMSA (gel migration assay) analyses both confirmed that G6P is the metabolic intermediate that activates the heterocomplex MondoA/Mlx to elicit the expression of Txnip. Additionally, the three-dimensional structure of MondoA is modeled, and the binding mode of G6P to MondoA is also predicted by *in silico* molecular docking and binding free energy calculation. Finally, free energy decomposition and mutational analyses suggest that certain residues in MondoA, GKL139-141 in particular, mediate its binding with G6P to activate MondoA, which signals the upregulation of the expression of Txnip.

## Introduction

Glucose is the preferred fuel for virtually all living organisms, and it can directly enter glycolysis. Cells take up glucose as a nutrient, which, on the one hand, provides a carbon skeleton for the cells, and on the other hand, supplies bioenergy for the synthesis of the ATP molecules. The imbalance of glucose metabolism in mammalian cells can cause metabolic disorders, and the most important feature of various cancers is disordered metabolism. At present, MondoA/Mlx and its downstream target gene Txnip have been proposed as a checkpoint for glucose metabolism, indicating that this transcription factor and the target gene are involved in sensing the glucose flux and inhibiting glucose absorption, which are very important for the homeostasis of glucose (Dupuis et al., 2010). Txnip can regulate the homeostasis of intracellular glucose and lipid metabolism and is also related to cell proliferation, migration, and apoptosis. Txnip can bind to the thioredoxin protein to change the intracellular redox status, and it is an endogenous inhibitor of thioredoxin protein (Patwari et al., 2006). In addition, *Txnip* is also called a tumor suppressor gene, although the underlying regulatory mechanism is still unclear.

Expression of the *Txnip* gene can be regulated by a number of factors. MondoA, which can dimerize with Mlx to bind with ChoRE (Wilde and Ayer, 2015), regulates the expression of Txnip (Richards et al., 2018). MondoA is mainly present in the cytoplasm on the outer membrane of mitochondria (Sans et al., 2006). MondoA is a very important transcription factor that regulates enzymes involved in the metabolism of most carbohydrates in the cell, and conversely, it can be regulated by the metabolic intermediates of carbohydrates. Both MondoA and Mlx contain the bHLHZIP domain (McConnell, 2016). The MondoA-Mlx complex can shuttle between the nucleus and cytoplasm in response of intracellular glucose levels (Sans et al., 2006). Accumulating in the nucleus, binding to the promoters, and recruiting other transcription factors, such as histone acetyltransferases and histone deacetylases, are the three steps needed for MondoA transactivation (Peterson et al., 2010). Many pioneering works have been performed on the structures and functions of MondoA, its cellular distribution, nuclear importation and exportation, etc. to determine how the glucose signal can be sensed by MondoA. Both MondoA and ChREBP contain conserved domains at the amino terminal of the sequences (Richards et al., 2017), called the Mondo Conserved Regions (MCRs) I-V. MCRs (Sillam-Dussès et al., 2016), which have been shown to be able to sense glucose, are also called the glucose-sensing element (GSM) (Teesalu, 2017). GMS contains low glucose suppression elements (LIDs) and glucose responsive activation conserved element (GRACE). The LIDs are the four conserved domains of the MCRs I-IV, inhibiting the activation of MondoA at low glucose concentrations, and MCR III provides the structural basis for transcriptional activation. GRACE, a conserved domain of MCR V, activates proteins upon receipt of a signal. It has been found that there is a reversible state of intermolecular mutual inhibition between the LIDs and GRACE in the GSM domain in ChREBP. High glucose can relieve the inhibition of GRACE by LIDs, which has directional sensitivity under external stimulation and reversibility (Li et al., 2006).

The effect of the regulation of Txnip on the flow of glucose is as below. Extracellular glucose enters the cell via a glucose transporter protein (Glut 1 and Glut 4) and is phosphorylated by the glycolytic kinase (HK) (John et al., 2011) in the glycolytic pathway to produce glucose 6-phosphate (G6P). G6P continues to be metabolized in glycolysis by PGI (phosphoglucose isomerase) and the phosphorylated pentose pathway by 6-phosphate glucose dehydrogenase (G6PD), an intermediate that activates MondoA/Mlx, which binds to the carbohydrate-sensing regions (ChoREs) on the *Txnip* promoter, and a set of MondoA/MLX dimers bind to one ChoRE. The transcription factor complex induces downstream Txnip protein expression. The expression of Txnip can in some way inhibit glucose uptake by the cells, thereby completing a negative feedback loop (Saha et al., 2018).

To explore how the expression of Txnip monitors glucose flux, we first explore the activation mechanism of MondoA/Mlx. More direct experimental verification is also urgently needed to explore the way in which MondoA/Mlx senses intracellular glucose flux. By combining experimental evidence (Hernandez-Guzman et al., 2003) and theoretical technologies, we modeled the 3D structure of MondoA and predicted the binding mode of G6P targeting MondoA. Furthermore, we mutated MondoA based on residues with critical potency in its binding with G6P. Previous studies (Li et al., 2006; Davies et al., 2010) have shown that some mutations of MondoA do not affect its dimerization with Mlx and its entry into/exit from the nucleus, but they have an effect on the binding of MondoA to the promoter. That is, the key amino acids involved in binding to G6P are affected, further affecting the activation of MondoA by G6P, thus demonstrating that the predicted and mutated amino acids hMondoA 139-141 GKL are key sites for G6P binding and that MondoA is activated by these sites to ensure its promoter binding and the transcription of downstream genes.

## Materials and Methods

### Cell Culture

HeLa, Bcap37, and 4T1 cells were cultured in an incubator containing 5% CO_2_ at 37°C using low and high glucose DMEM, with 10% FBS and 1% penicillin-streptomycin, respectively. Cell starvation was generated with glucose-free DMEM supplemented with 10% FBS and 1% penicillin-streptomycin and 2 mM pyruvate sodium.

### Cloning the Plasmids

The eukaryotic expression plasmid vector was Pxj40 with Flag or HA tags. Vectors expressing MondoA and Mlx with Flag and HA were used as preservation materials for the laboratory in mammalian cells. The mutations of MondoA were constructed using a one-step cloning kit from Vazyme (C211-01).

### RNA Extraction, RT-PCR and QPCR

RNA was extracted using an RNA extraction kit from Axygen. And Invitrogen kit was used for reverse transcription. Real-time quantitative PCR was performed using the KAPA fluorescence quantification kit.

### SDS-PAGE and Western Blot

The antibodies used in WB were tubulin from Huaan (M1305-2), and Txnip from Abcam (ab188865); MondoA from Abcam (ab77294); Flag from Yisheng (30503ES60); RNA polymers II from Milipore (05623B); and GAPDH, which are homemade antibodies with high efficiency.

### Immunofluorescence (IF)

The cells were washed and fixed with 3% para. For antibodies, anti-Flag antibody (Yisheng) was used, and the nucleus was stained with Hochest 342224 and incubated with the secondary antibody. Using the Olympus V3000 microscope at 60× oil magnification, the fluorescence intensity and colocalization at 488 and 546 nm were examined.

### Dual Luciferase Assay

The Promega Dual Luciferase Assay Kit was dissolved at room temperature in advance. Lysing solution was used for 15 min at room temperature. The luciferin substrate was added to measure the chemiluminescence L1, and then the substrate for Renilla was added to perform the chemiluminescence measurement L2. For the calculation, L1/L2 is used to remove the internal reference.

### Gel Migration Assay (EMSA)

#### Reaction System

Following the protocol of Thermo EMSA Kit (18890) for the reaction system.

#### Probes

Labeled probes and unlabeled probes were designed, and the labeled probe had a biotin label on the 5' end. The probe sequence was designed based on the MondoA binding region in the Txnip promoter region (F: GGGATGTGCACGAGGGCAGCACGAGCCT CCGGG, R: CCCGGAGGCTCGTGCTGCCCTGTCGTGCACATCCC).

#### Running the Gel

A 4% non-reducing PAGE gel (without SDS) was used. A 16-mA current was run for 30 min, and bromophenol blue went to 1/3 of the bottom of the gel. Then, the membrane was transferred for 45 min at 100 V and 380 mA. And HRP reacted with the substrate ECL to fluoresce the labeled probe.

### siRNA Knockdown

Design the siRNA by siDesign website. Transfection was performed when the cells were at ~50–60% confluence. The transfection reagent RNAi MAX and siRNA were mixed gently and added to the plate. After 4–6 h, 3-fold serum medium was added per well. The nucleotides sequences of the siRNA for glucose-6-phosphate dehydrogenase (siG6PD) and phosphoglucose isomerase (siPGI) are GCAAGGAGAUGGUGCAGAA, and is AGUCCAGGGGCGUGGAGGC.

### Nuclear Fraction Extraction

#### Cytoplasmic Lysation

After obtaining adherent cultured cells, the medium was discarded, and the cells were rinsed twice with cold PBS. Then, TNE buffer, PI and DTT were added, and a cell scraper was used to scrape gently in one direction to collect the cells, and the pellet was obtained after centrifugation at 1,200 rpm for 1 min. Four volumes of precipitated HB buffer with PI and DTT were added, suspension was achieved with a gun, and the cell membranes were lysed with a homogenizer that still retained the integrity of the nucleus and homogenate 10 times, after which it was centrifuged again to obtain a pellet. The supernatant was the cytoplasmic lysate (Cf).

#### Nuclear Extraction

The collected pellet (i.e., nucleus) was fragmented with a buffer containing a medium (0.25–0.30 M KCL) salt to dissociate most of the transcription factors, RNA polymerase and essential transcription factors and cofactors from the chromatin (the high KCL concentration is not sufficient to release histones). Three volumes of BC240 buffer (now PI and DTT) were added and resuspended for 1 h, with vortexing for every 5 min. The supernatant after high-speed centrifugation was the desired nuclear extract. The supernatant was removed and stored in a negative 80°C refrigerator after freezing in liquid nitrogen. The supernatant was quickly thawed before use.

### Chromatin Immunoprecipitation

#### Cross-Linking

Thirty-seven percentage formaldehyde (280 μl) was added to 10 cm diameter plate, mixed well at R.T. for 10 min. 1 ml of 1 M glycine was added and stayed for 5 min to stop. The cells were washed three times with cold PBS and harvested by scraping.

#### Ultrasonic

Add lysis buffer (containing 1% SDS, 25 mM EDTA, 50 mM Tris-HCl) to the cells and lysed by ultrasound with a non-contact cellular sonicator (200 μl/tube for 30 s) for 30 times. The cells were centrifuged and the supernatant was stored at −80°C.

#### IP (Immunoprecipitation)

Add nuclear lysate to ChIP diluent at a 1:10 ratio, with antibodies and magnet beads, rotated at 4°C overnight. Then the beads were washed four times in sequence with low salt buffer, high salt buffer, LiCl buffer, and TE buffer, with rotation at 4°C for 5 min between each wash.

#### De-Cross-Linking

Appropriate amount of elution solution (containing 1% SDS, 0.1 M NaHCO_3_) and Proteinase K were added to the beads, and the mixture was shaken at 65°C for 1–2 hr. After removal from the shaker, the solution was heated at 95°C for 10 min to remove the cross-linkage.

#### QPCR

The DNA was purified according to the kit instructions, and an appropriate amount of sample was removed to perform real-time PCR (e.g., Txnip) for the gene of interest (Txnip e-box primers, F: AGGTTTTAGGGTCAGTGGGAT, R: CTGCCCGGTCCTTGTTTAC; human HK2 e-box primers, F: GCCCCGCAGGTAGTCAGG, R: GACCACGATTCTCTCCACG; Negative control primers: F: ATGGTTGCCACTGGGGATCT, R: TGCCAAAGCCTAGGGGAAGA).

### Molecular Modeling Analyses

#### Homology Modeling

As mentioned above, to date, there is no crystal structure of MondoA available in the PDB database. Thus, before investigating the binding mechanism between MondoA and G6P, we first modeled the 3D structure of the protein. Based on McFerrin's pioneering work in modeling the structure of MondoA (McFerrin and Atchley, 2012), we also used human estrone sulfatase (PDB code: 1P49) (Hernandez-Guzman et al., 2003) as the template for the construction of MondoA, which shows a similarity of 27.6% with the sequence of MondoA. Modeler 9.20 (Ichiye and Karplus, 1991) was employed for the system modeling, and the best model (with the lowest global energy) was chosen for the following study.

#### Molecular Mechanics Minimization and MD Simulation

To relax the potential unfavorable local contacts between atoms of the modeled structure, the widely used four-step minimization and three-step MD simulation procedures were employed for structural optimization and system equilibrium, respectively (Sun et al., 2017). Here, the ff14SB force field was used for the protein parameterization (Maier et al., 2015). In the MD simulation, a 15-ns production run was carried out to equilibrate the system, and the final structure was used for the following study.

#### Molecular Docking

Autodock 4.2 (Morris et al., 2009) in conjunction with the Lamarckian genetic algorithm (LGA) (Morris et al., 1998) was used for ligand docking (including G6P and D2-G6P). Here, the binding pocket was set at S287 of MondoA as proposed in a previous publication (McFerrin and Atchley, 2012). The docking box was set at 50 × 50 × 50 grids to supply sufficient sampling space for the binding mode searching of the ligands. Each ligand was docked 100 times, and the best docking pose (with the strongest binding affinity) for each ligand was used for the following study.

#### Binding Free Energy Calculation and Decomposition

The best docked pose in each structure was employed for the end-point binding free energy calculation. Before calculating the binding free energy, the structures of the ligand-receptor complexes (including the wild-type MondoA and the mutants) were reconstructed by the *tleap* (Wang et al., 2006) module in the amber14 package. Here, the general amber force field (*gaff*) (Wang et al., 2004) was used to parameterize the small-molecule ligands. AM1-BCC charge (Jakalian et al., 2002) was employed as the atomic charge for the ligands. All the complexes were reoptimized with the four-step minimization protocol as mentioned above.

The Molecular Mechanics/Generalized Born Surface Area approach (MM/GBSA) was used for the binding free energy calculation and decomposition (Kollman et al., 2000; Hou et al., 2011; Sun et al., 2016). Here, only the optimized structures were employed for the MM/GBSA calculations because the minimized structures usually give a better results than that based on other MD structures (Xu et al., 2013; Sun et al., 2014a,b). All MM/GBSA calculations were performed with the *MMPBSA.py* module (Miller et al., 2012) in amber14 using the default parameters (Sun et al., 2013, 2014). It should be noted that, here, no entropy part was incorporated in the MM/GBSA calculations, and thus the calculated binding free energies seem too large compared with the common-level of the wet experiments (~-10 kcal/mol). Nevertheless, here we only want to characterize the delta values between the wild-type and the mutated MondoAs, and we would like to call the current binding affinities “effective binding free energies” as in other's work.

## Results

### Correlation Between Txnip Expression and Intracellular Glucose Concentration

**Figure 1:**
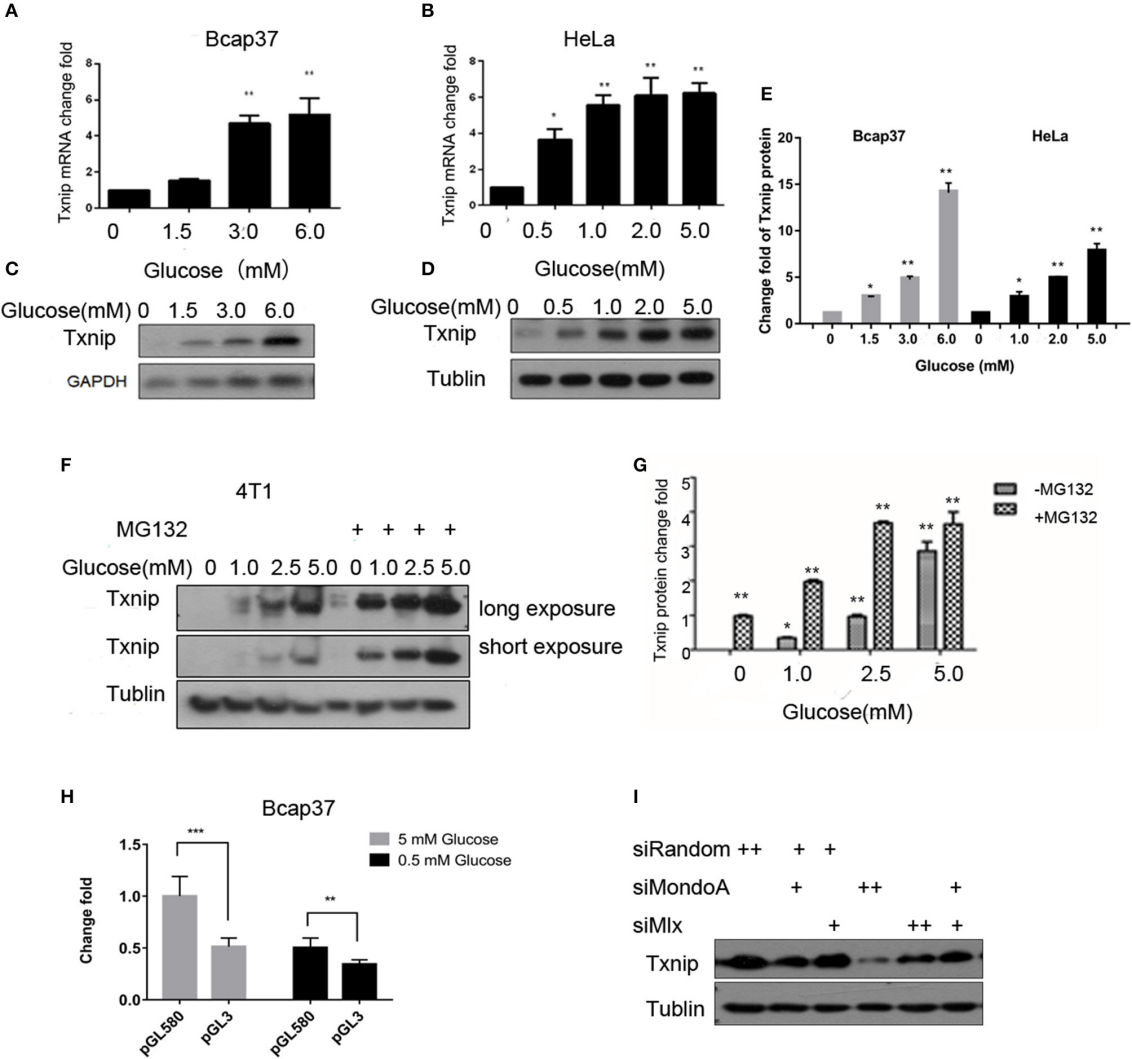
The expression of Txnip is significantly positively correlated with the intracellular glucose levels. **(A,B)** mRNA level of Txnip expressed in the human breast cancer Bcap37 and HeLa cell lines at the indicated glucose concentrations (*T*-test, *P < 0.05; **P < 0.01; ***P < 0.001); **(A)** Bcap37; **(B)** HeLa; **(C,D)** protein level of Txnip expressed in the human breast cancer Bcap37 and HeLa cell lines at the indicated glucose concentrations; **(C)** Bcap37; **(D)** HeLa; **(E)** quantification of Txnip protein expression level at the indicated glucose concentrations in Bcap37 and HeLa (*T*-test, *P < 0.05; **P < 0.01; ***P < 0.001); **(F)** protein levels of Txnip expressed by the mouse breast cancer 4T1 cell line at the indicated glucose concentrations with or without MG132; **(G)** quantification of Txnip protein expression level at the indicated glucose concentrations in 4T1 (*T*-test, *P < 0.05; **P < 0.01; ***P < 0.001); **(H)** double luciferase reporter gene test of the binding efficiency of MondoA to the Txnip promoter at different glucose conditions (*T*-test, *P < 0.05; **P < 0.01; ***P < 0.001); **(I)** Txnip expression on the condition of knockdown MondoA and Mlx.

Txnip is an important protein that maintains glucose homeostasis and regulates glucose metabolism. The expression of Txnip in cells is closely related to the concentration of intracellular glucose. When cells are starved for 4 h in serum-free and glucose-free medium, they can consume intracellular glucose to a very low concentration. Thus, we treated the cells in different glucose concentrations together with/without MG132 for 4 h, we used several cell lines, such as HeLa, 4T1, HepG2, 293A, HL7702, RKO, C2C12, and HUVEC, to test the relationship between the glucose concentration and the expression of Txnip, and we observed the same expression patterns. As shown in Figure 1, the Txnip expression in the Bcap37 and HeLa cell lines significantly increased at both the mRNA and protein levels (Figures 1A–D) along with the elevating glucose concentration after 4 h treatment, grossly substantial increase can be seen after quantification (Figure 1E). There was also a significant positive correlation between the glucose levels and Txnip expression in 4T1 cells (Figures 1F,G), and Figure 1G show that the degradation of Txnip is mediated by protease. The elevated expression of Txnip inhibits the absorption of glucose by cells via a certain mechanism, thus ensuring intracellular glucose homeostasis, which has also been observed in other previous works (Wu et al., 2013; Waldhart et al., 2017) on Txnip-regulating glucose transporters (Rumsey et al., 1997) (Glut 1 and Glut 4), thus ensuring glucose homeostasis in cells.

The promoter of Txnip has many cis-acting elements. The glucose-sensing element is a carbohydrate-sensing element (ChoRE), and the transcription factor Mondo/Mlx bound to the ChoREs. MondoA is a main transcription factor of Txnip, and the expression of Txnip depends on the amount of activated MondoA. To test this, we do the following experiments. We constructed a pGL3 luciferase reporter plasmid containing ChoREs where MondoA binds to the Txnip promoter. Plasmids with Txnip promoter cloned in the pGL3 vector-Txnip promoter 580 (TSS), MondoA wild-type, Mlx, and pRL-TK were transfected into cells. The results showed that the wild-type MondoA/Mlx has a low level of activation of the reporter plasmid in low glucose concentrations (0.5 mM glucose). And the activity was significantly higher at high glucose concentrations (5 mM glucose) than that in the low glucose environment (Figure 1H). This indicates that the amount of MondoA binding to the luciferase reporter gene plasmid is also significantly increased at a higher glucose concentration, when the glucose is sufficient. We also conduct the knockdown assay of MondoA and Mlx (Yu et al., 2009) in 5 mM glucose DMEM medium. And the result showed a sharp decrease of Txnip expression (Figure 1I), which proves MondoA is strictly related to the expression of Txnip. In summary of the above, glucose level is a sufficient condition for MondoA activation, and the binding of activated MondoA to ChoREs is another sufficient condition.

With regard to Txnip, MondoA is both an activating transcription factor and an inhibitory transcription factor, depending on the state of the cell. When the intracellular glucose concentration is appropriate or high, the cells mainly rely on glucose oxidation (Small et al., 2018) to provide energy. And when the metabolic flux (Wegner et al., 2015) reaches up to a certain level, MondoA is activated, which in turn activates the Txnip promoter to promote its expression. However, when the intracellular glucose content is low and the cells need to use glutamine metabolism to provide energy, MondoA inhibits the expression of Txnip under the influence of the mTOR pathway (Kaadige et al., 2009; Noordeen et al., 2010; Boergesen et al., 2011). The scope of this research focuses on the first circumstance. In the normal blood supply state of the tissue, the blood glucose concentration is ~5.0 mM, and the tumor tissue has vascular malformations (Feng et al., 2016). The blood supply is insufficient, and in particular, the blood supply inside the tumor is very poor, as it is only approximately one-tenth of the normal blood supply (~0.5 mM). In contrast, *in vitro* cultured cell-lined tumor tissue, the glucose supply is sufficient, and there is a sufficient amount of MondoA freely shuttling between the cytoplasm and nucleus to sense glucose metabolism flux and then activate the expression of Txnip.

### Glucose Metabolic Intermediate Promotes the Expression of Txnip

**Figure 2:**
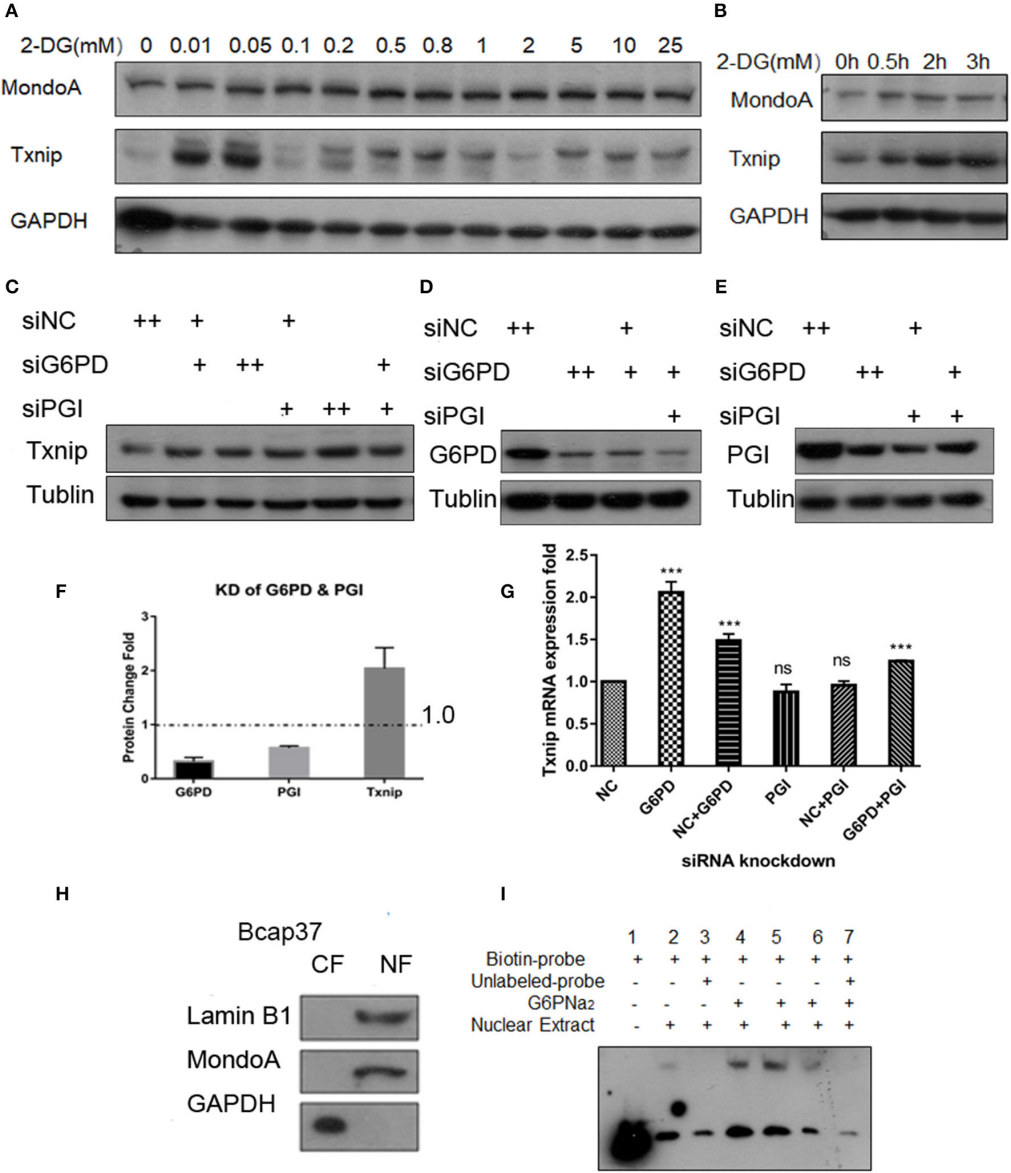
G6P accumulation can increase the expression of Txnip. **(A)** Changes in expression of Txnip after treating with 2-DG 0–25 mM; **(B)** The expression of Txnip and its transcription factor MondoA in 2 h time scale treated by 200 μM 2-DG; **(C,G)** The protein and mRNA expression levels of Txnip after knockdown of two catalytic enzymes (PGI and G6PD) (*T*-test, *P < 0.05; **P < 0.01; ***P < 0.001); **(D,E)** The protein levels of Txnip after knockdown of two catalytic enzymes (PGI and G6PD); **(F)** The quantification amount of G6PD, PGI, and Txnip expression in HeLa cells after knockdown PGI and G6PD; **(H)** Separation efficiency of the nuclear isolation of cultured Bcap37 cells; **(I)** MondoA binding efficiency in the EMSA experiment increased with the different concentrations of G6PNa_2_ added to the reaction system.

2DG is quite a common proxy of glucose used to test the glycolysis flux (TeSlaa and Teitell, 2014). Some proofs from published papers (Li et al., 2010; Peterson et al., 2010) show that the glucose analog 2-DG is used to produce 2D-G6P, the analog of G6P, which accumulated in cells and induces Txnip expression. Our experiments show that the addition of 0.01–25 mM 2-DG, Txnip expression increase in comparation with control (Figure 2A) in Bcap37 cell lines, although HeLa and 293A show different increasing pattern of Txnip after 2-DG treatment, all are elevated by 2-DG (Figures S2A,B). And in a 3 h time scale, the expression of Txnip is induced along with the accumulation of 2D-G6P and the expression of MondoA stays steady in a certain level (Figure 2B), this provides us the activation of transcription factors depend on the cellular G6P level.

To assess the relationship of G6P and the regulation of Txnip-MondoA/Mlx axi, we focused on G6P and the following experiments were performed. First, we designed siRNA for the two key enzymes, PGI and G6PD, and conducted knockdown experiments with the Bcap37 and HeLa cell lines. After simultaneously knocking down PGI and G6PD (Figures 2D,E), G6P accumulated in the cells, and at the same time, the expression of Txnip was detected. As shown in Figures 2C,F, the protein level of Txnip increased in all the knockdown groups, and it also showed that the mRNA levels of Txnip also increased in G6PD knockdown group (Figure 2G). These results indicate that the effect of endogenous accumulation of G6P is potent in inducing the expression of Txnip.

Importantly, to confirm the activating effect of G6P on MondoA, we also used gel migration assay (EMSA) to detect the binding effect of MondoA according to the principle that the activated transcription factor is still able to bind to its promoters *in vitro* in the buffer system provided. EMSA is a classic method for displaying transcription factors binding *in vitro* to downstream promoters, and here we used it to reflect the activation of the small molecule G6P on the transcription factor MondoA/Mlx. The biotin probes were designed for the promoter of MondoA-conjugated Txnip ChoREs, and different concentrations of G6P were added as activators in the reaction system.

After 10 mM glucose treatment of Bcap37 for 4 hr, we separated the cytosolic and nuclear fractions and the two components were detected by WB (Figure 2H), and protein quantification was performed with a BCA standard curve for EMSA. As shown in Figure 2H, the effect of nucleo-plasmic separation was detected by LaminB1 and the cytosolic protein GAPDH proteins in the nucleus. In the EMSA reaction system, G6PNa_2_ was added as the source of G6P. And we tested the amount of transcription factor MondoA/Mlx bound to the ChoRE probes. The results showed that to some degree, more MondoA/Mlx was bound in higher G6P concentration group. It can be seen from Figure 2I that the binding of MondoA to the promoter probe was significantly promoted by the activation of G6PNa_2_ and increased with elevated G6PNa_2_ concentration, such as in lanes 4 and 5, which show much higher concentrations than the treatment group without G6PNa_2_ (lane 2). However, when the concentration of G6PNa_2_ continued to increase, the binding of MondoA to the probe decreased; see lane 6. As the concentration increased to 500 nM, the activation level disappeared. The reduction in activation may be explained by the threshold of MondoA activation, excessive G6P stimulation will depolymerize the complex, or it may be that a high ion concentration will affect the stability of the reaction system. Nevertheless, further experiments are needed to detect the specific reasons. Clearly, we can see that both 0.05 and 0.25 mM G6PNa_2_ elevated the binding proteins, which means that the activation level of MondoA is proportional to the concentration of G6P within a certain concentration range. Therefore, G6P can elevate the activation of MondoA both *in vivo* and *in vitro*. Next, we wanted to determine the binding mechanism of G6P and MondoA with further efforts.

### Binding Mechanism Between G6P and MondoA

**Figure 3:**
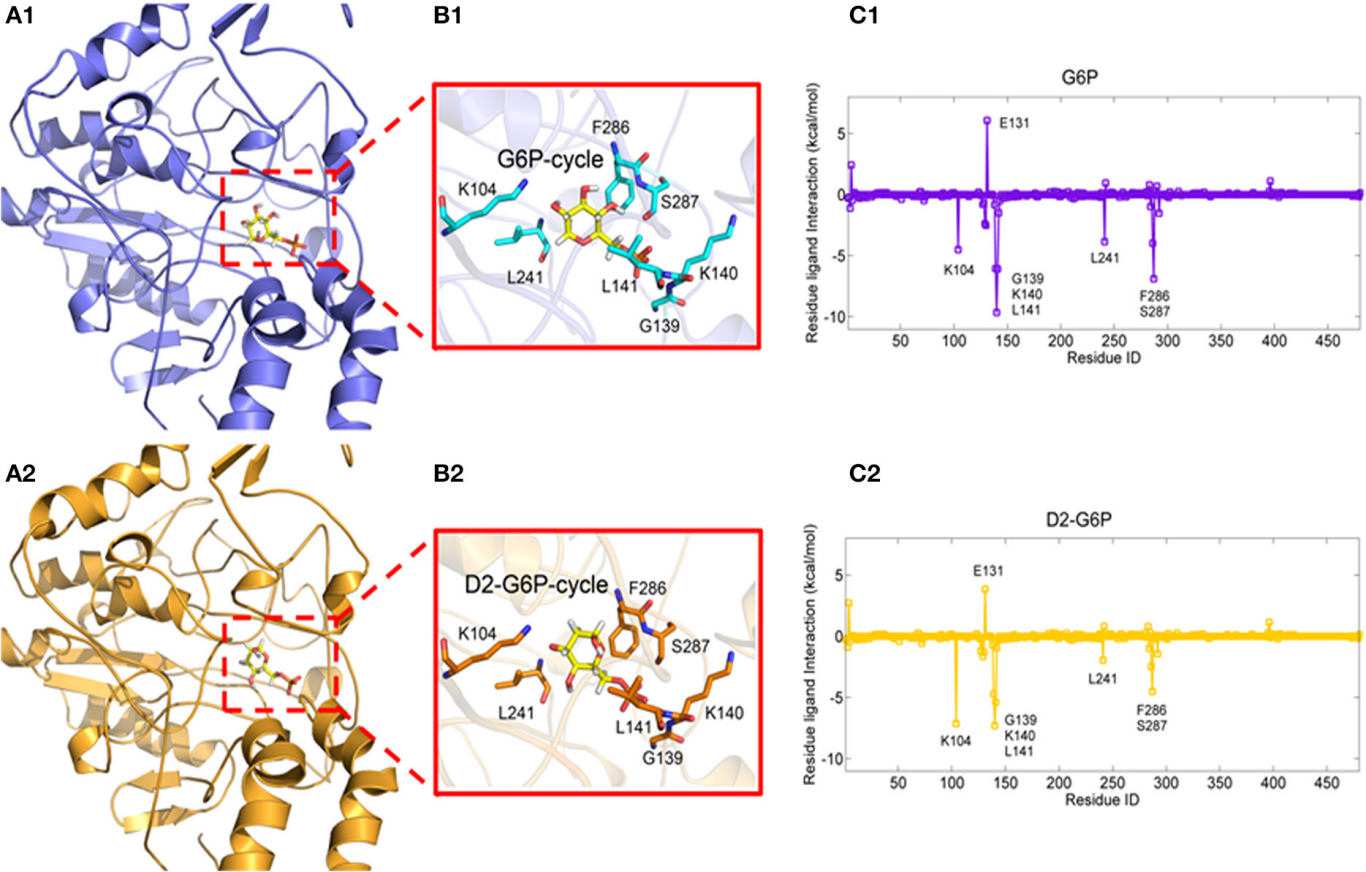
Complexes of MondoA binding with G6P (**A1**, yellow stick model) and D2-G6P (**A2**, yellow stick model). The top contributing residues are derived from the corresponding residue-ligand interaction spectra (**C1, C2**, predicted by MM/GBSA energy decomposition) shown in the purple and orange stick models in **B1, B2**, respectively.

To explore the molecular mechanism of G6P-induced activation of MondoA, we conducted a series of *in silico* analyses. Because there are no crystal structures of MondoA available in the PDB database, we first modeled the 3D structure of MondoA based on McFerrin's work (McFerrin and Atchley, 2012) (Figure S3a). After that, we performed a 15-ns molecular dynamics (MD) simulation to equilibrate the system, in which the system reached equilibrium rapidly within 3 ns with the RMSD stably fluctuating around 5.5 Å (Figure S3b), indicating that the system is stable and suitable for the following analyses. Thus, G6P was docked into the proposed active site of MondoA using the equilibrated MD conformation (Figures 3A1, A2). To provide a comparison, the G6P analog, D2-G6P, was also considered in the docking study. As shown in Figures 3B1, B2, both docked ligands (G6P and D2-G6P, yellow bar model) showed a similar binding pose to MondoA, meaning that the docking pose of the top-scored conformation was reasonable for the following analysis. Therefore, we conducted MM/GBSA free energy decomposition analysis (Genheden and Ryde, 2015) to detect the vital residues for the binding of the small molecules. As shown in Figures 3C1, C2, both ligands show two important binding regions on MondoA, which includes the S287 region proposed in McFerrin's work (McFerrin and Atchley, 2012) and the newly discovered GKL139-141 region identified in our own simulation. Both regions contribute more than 5 kcal/mol to the ligand binding. The conserved residues at positions 286-287 (F286 and S287, as shown in Figures 3C1, C2) are present in the six conserved residues of MCR6, as mentioned above. A structural view of the binding mechanism of the two ligands shows that these residues (the cyan/orange stick models in B1 and B2 in Figure 3) are located very close to the ligands (the yellow stick models in B1 and B2 in Figure 3), meaning that these residues may contribute the most to the binding of the ligands.

To further validate the residues important for binding with the ligands, virtual mutations were introduced into wild-type MondoA using VMD software (Humphrey et al., 1996). Here, the binding states of MondoAs (G6P-MondoA and D2-G6P-MondoA) were used as the initial structure for the mutations. The mutations studied here were S282G, T284A, S287A, GKL139-141GAA, and DEL139-141. All the mutated systems were reminimized for 20,000 steps to relax the local unfavorable contacts. The MM/GBSA free energy calculations were then performed for all systems, and the final results are summarized in Table 1.

**Table 1:** Prediction of the free energy of small molecule G6P and D2-G6P binding (kcal/mol) by wild-type and mutant MondoA.

**Mutants**	**G6P**	**D2-G6P**
WT	−38.25	−16.35
GKL139-141GAA	−33.13	−13.00
DEL139-141	−21.99	−0.78
S282G	−38.47	−16.67
S287A	−33.17	−14.29
T284A	−36.54	−16.18

As shown in Table 1, for the wild-type MondoA, the binding free energy of G6P is significantly higher than that of D2-G6P (−38.25 vs. −16.35 kcal/mol), indicating that binding with endogenous G6P is more favorable for MondoA, which is consistent with evolutionary selection pressure. Moreover, similar binding behaviors are shown for G6P and D2-G6P for all the investigated MondoA mutants compared with those in the corresponding wild-type MondoA. For example, significantly reduced binding affinities are shown in the GKL139-141 deletion MondoA (−21.99 vs. −38.25 kcal/mol for G6P; and −0.78 vs. −16.35 kcal/mol for D2-G6P) and the GKL139-141GAA mutated MondoA (−33.13 vs. −38.25 kcal/mol for G6P; and −13.00 vs. −16.35 for D2-G6P) for both G6P and D2-G6P, meaning that the proposed regions may make important contributions to ligand binding (this has been partly validated by our experiments as shown in the next section, implying that the current predictions are reasonable).

### Effects of MondoA and Its Mutants on Txnip Expression

As predicted by our *in silico* model in the last section, several residues play vital roles in binding with the small molecules G6P and D2-G6P. To further validate our results, we mutated/deleted the abovementioned residues in MondoA and constructed mutant plasmids. The mutations of MondoA are distributed in the regions of MCRII, MCRIII, and MCR6.

**Figure 4:**
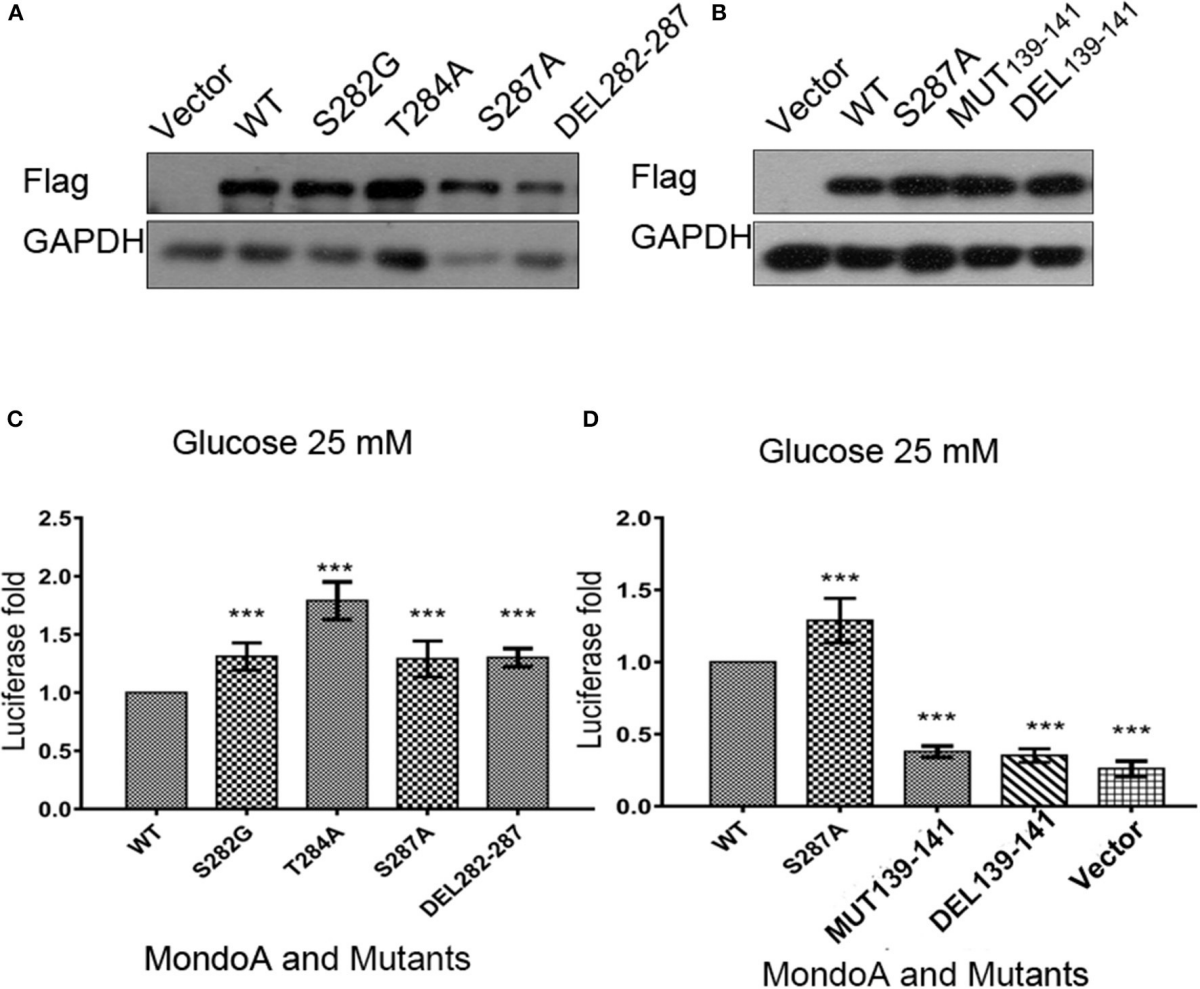
The effects of several key point mutations on MondoA on the Txnip promoter. **(A,B)** The cells were transfected with mutant plasmids S282G, T284A, S287A, DEL282-287 and 139-141 mut, DEL39-141, and the wild-type plasmid for overexpression testing (Flag tag). **(A)** Vector, WT, S282A, T284A, S287A, and DEL282-287, **(B)** Vector, WT, S287A, 139-141GAA, and DEL139-141; **(C,D)** Detection of MondoA activity by the dual luciferase reporter gene assay under 25 mM glucose for 4 h (*T*-test, *P < 0.05; **P < 0.01; ***P < 0.001). **(C)** WT, S282A, T284A, S287A, and DEL282-287; **(D)** WT, S287A, 139-141GAA, DEL139-141, and Flag-vector.

For the six conserved amino acids (282 SDTLFS) human MondoA (mRNA 2760 bp) in the six MCRs proposed in the literature, we mutated the acidic amino acids 282S, 284T, and 287S, respectively, i.e., S282A, T284A, S287A, as well as the mutation DEL282-287 with six amino acids missing. Based on bioinformatics and our own MondoA model for small molecule docking experiments, we also constructed the mutants GKL139-141GAA and DEL139-141 MondoA. These plasmids were used in the protein expression experiments. The effects of these mutants on Txnip expression were detected by the dual luciferase reporter gene using a pair of intact ChoREs and biotinylated oligonucleotide fragments. The constructed MondoA series of plasmids, Mlx-HA plasmid, and pGL580 which contains the promoter sequence of Txnip intact ChoREs, and the plasmid pRL-TK of the sea kidney as an expression control over the plasmids are for the transfection experiments. The efficiency of plasmid expression was all good and almost in the same levels in consideration to the control GADPH (Figures 4A,B).

From the results of the dual luciferase reporter gene assay (Figures 4C,D), we can see that luciferase level of the mutants of GKL139-141GAA and DEL139-141 are very close to that of the control vector group, indicating that mutations at residues 139-141 in MondoA are somehow in great possibility vital for its binding on the promoter for transcription.

### Subcellular Distribution of MondoA Mutant

**Figure 5:**
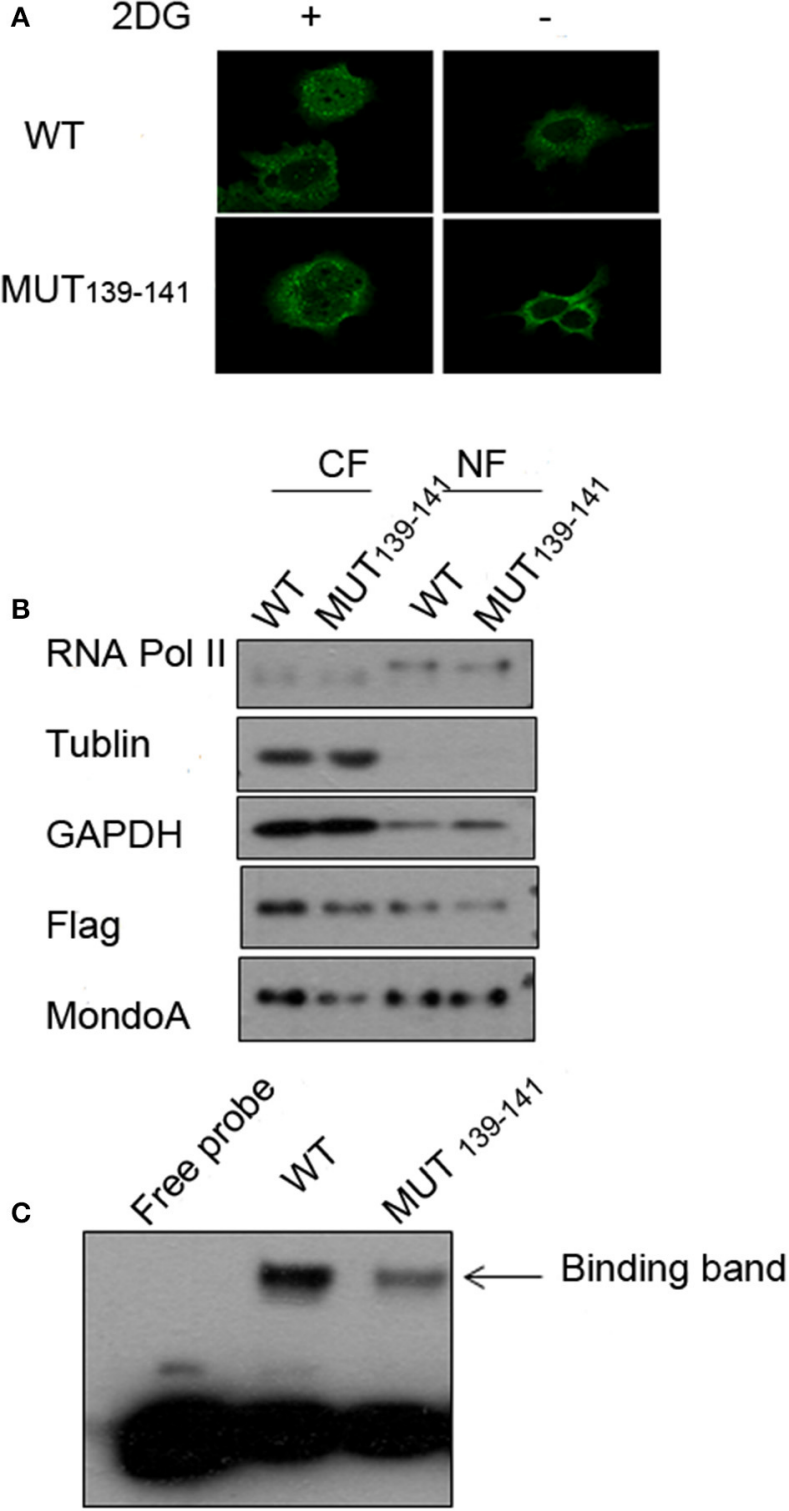
Several key point mutations of MondoA have decreased occupation on the Txnip promoter. **(A)** The distribution of wild type (WT) and mutant (139-141 mut) MondoA in the cell w/o 2DG stimulation; **(B)** Nuclear separation with regard to the distribution; **(C)** The binding efficiency of wild-type (WT) and mutant (139-141 mut) Mondo A in the EMSA (gel shift) test.

Because the mutations of residues 139-141 in MondoA can seriously affect its binding rate to Txnip promoter, we wonder that is the nuclear-entering process of MondoA affected by the mutations, or is the mutant MondoA cannot be activated by G6P at all? To answer this question, we performed immunofluorescence (IF) and nuclear separation experiments for GKL139-141GAA-mutated MondoA to determine whether the subcellular distribution of MondoA was changed after mutation. To detect the distribution of MondoA GKL139-141GAA in Bcap37 cells, wild-type MondoA-Flag and GKL139-141GAA-Flag with its heterodimer Mlx-HA were overexpressed and followed by culturing in glucose-free medium and 2DG for 4 h, respectively. And the CRM1-dependent nucleation inhibitor LMB was added. From Figure 5A, it can be seen that the green fluorescent staining of the Flag tag fusion protein was widely distributed under the 60-fold oil microscope, demonstrating MondoA distribution in both the cytoplasm and nucleus. 2DG-stimulated and non-stimulated MondoA have different distribution ratios inside and outside the nucleus. The cells added to the 2DG treatment showed a significant increase in the nuclear concentrations of the wild-type and mutant MondoA proteins, as reported by several articles (Stoltzman et al., 2008; Havula and Hietakangas, 2012), indicating that the mutation did not affect the entrance of the Flag-tagged MondoA proteins into the nucleus. The results showed that the nuclear entry of the mutant MondoA was not significantly different from that of the wild type, indicating that the mutation had no structurally significant effect on the mutant.

We also quantified the distributions of exogenous MondoA in the cytoplasm and nucleus by performing nuclear separation tests. From the results of WB, the amounts of the internal reference tublin and GAPDH are very close, and the amount of the mutant in the cytoplasm is lower than that of the wild type. This may be attributed to the transfection efficiency of the mutant, but it did not affect the amount of MondoA in nucleus. The probability of the wild-type MondoA/Mlx and mutant GKL139-141GAA MondoA/Mlx entering into the nucleus was almost the same (Figure 5B). After quantification, entry rate of wild-type was ~30%, and the mutant was ~40%. Many articles (Eilers et al., 2002; Davies et al., 2008; Peterson et al., 2010) have found the intramolecular elements NES and NIS have the ability to affect the access of MondoA to the nucleus by analyzing the function of the secondary structure of MondoA and performing mutant experiments regarding protein entry and exit into the nucleus. Therefore, the mutations at hMondoA 139-141 are different from some highly conserved site mutations. Mutations at these three sites (139-141) did not affect the entry of MondoA into the nucleus (Figures 5A,B). In conclusion, the results of the nuclear separation and immunofluorescence experiments showed that the nuclear level of the MondoA mutant was basically consistent with that of the wild type.

To further investigate the effects of MondoA mutation on the behavior of the downstream signaling pathway, we examined the binding of MondoA in the nucleus to the promoter of Txnip by gel migration experiments (EMSA). With equivalent protein of the exogenous wild type and mutant GKL39-141GAA MondoA, the binding amount of the wild type to the promoter was significantly higher than that of the mutant (Figure 5C), although the expressions of the Flag fusion proteins are consistent (Figure 5B). We believed that the non-sense mutation at 139-141 site of MondoA caused conformation changes in the 3-dimention structure, preventing it from being activated by G6P in the cell. Compared to the wild type, the binding rate of the 139-141 mutant on the promoter was significantly reduced, and the slight combined band might be contributed by the endogenous MondoA protein in the cells, which indicates that the mutant cannot activated by G6P (Figure 5C).

### Effect of MondoA and Its Mutants on the Expression of Related Genes Containing ChoREs

**Figure 6:**
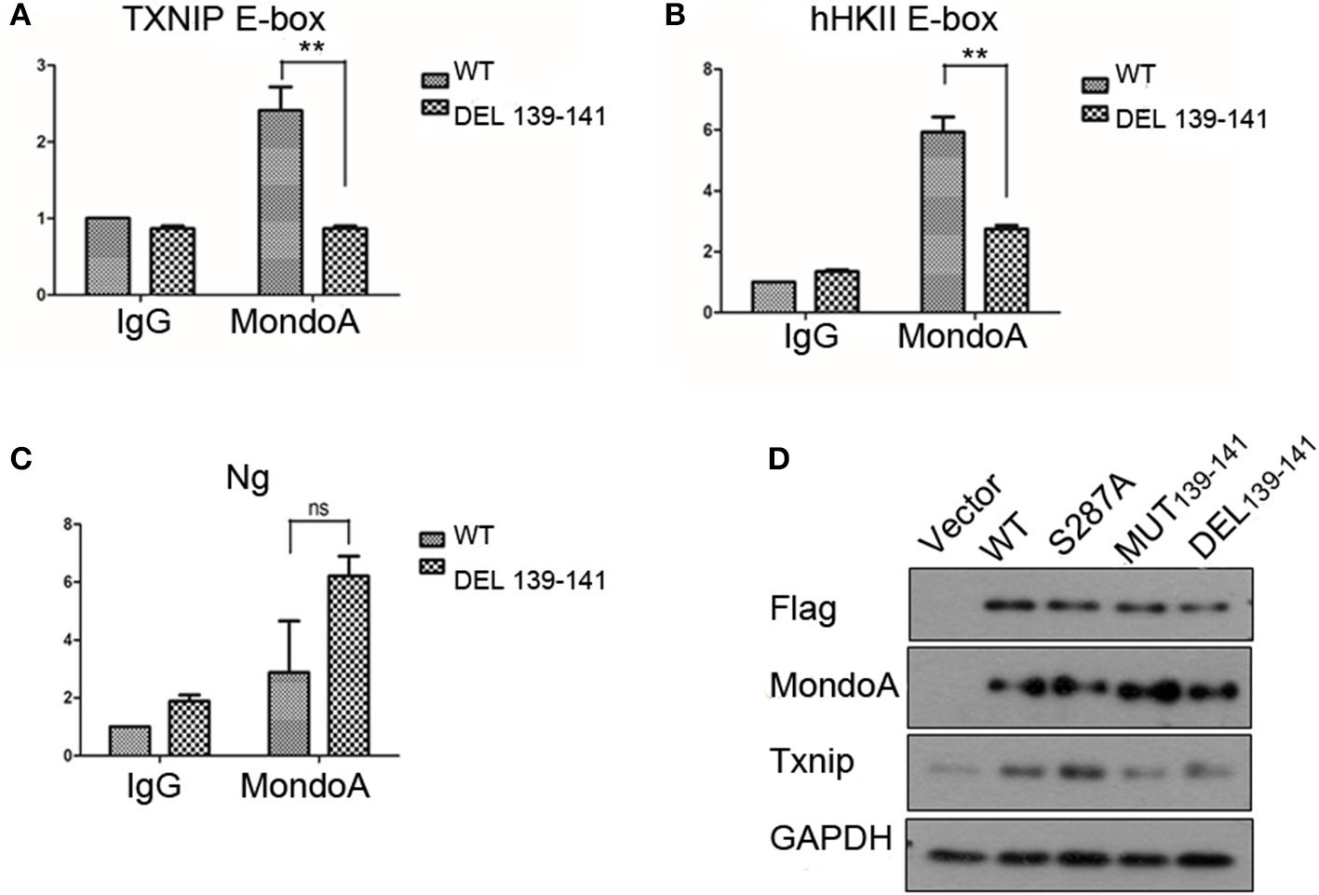
The binding rate of the wild-type and mutant MondoAs to the downstream gene promoter are different **(A–C)** (*T*-test, *P < 0.05; **P < 0.01; ***P < 0.001) and so is the expression level of Txnip protein. **(A)** The binding rate of MondoAs to the Txnip promoter E-box; **(B)** the binding rate to the hHkII promoter E-box; **(C)** the binding rate to Ng (negative control); **(D)** Txnip protein expression after WT or mutant plasmid overexpression.

As a transcription factor, MondoA controls multiple genes that are involved in carbohydrate metabolism, and MondoA/Mlx is mainly responsible for 75% of the gene expression in the glycolytic pathway (Li et al., 2006). The more representative ones are Txnip and hHKII. In our experiments with the wild-type and mutant MondoA binding to the promoter *in vitro*, we showed that the mutant could not be activated by G6P. We wanted to detect cells transfected with both wild-type and mutant MondoA plasmids and to determine how the expressions of Txnip and hHKII were affected. After 139-141 is mutated, does it reduce the binding to most target genes? We studied the occupation rate of Txnip and HKII by MondoA/Mlx ChoREs. Using the chromatin immunoprecipitation technique (ChIP), nucleic acids were extracted that constituted the downstream promoter fragment of the wild-type and mutant MondoA, and they were measured by QPCR, and the results are consistent with our prediction. Compared to that of the wild type, the effect of GKL39-141GAA MondoA mutant on downstream gene expression was quite less, nearly as slight as that of IgG. The results are shown in Figures 5A,B. The probabilities of MondoAs (wild type and mutant) binding to the Txnip promoter were ~8 and 0.3%, respectively, while to hHKII promoter were ~3 and 0.01%, respectively (Figures 6A,B). Therefore, the mutant cannot be activated by a high concentration of G6P in the cell and cannot efficiently bind to the downstream promoters. We take the human Ng as a negative control, which targeted gene desert on chromosome 12 (Figure 6C).

Similarly, we performed Western blot experiments on Txnip expression in several specific mutants. After transfection for 20 h, the glucose-free medium cultured for 2 h and then replaced with 10 mM glucose in DMEM for 4 h. And the expression of the downstream protein Txnip was detected. The mutant plasmid S287A was chosen because it is one of the six amino acids in McFerrin's work (McFerrin and Atchley, 2012) and plays a role in our docking model; meanwhile, S287 contributes to the energy change after the non-sense mutation. MondoA was measured both by flag tag and MondoA itself. And only one MondoA band in ectopic expression shows that the amount of endogenous MondoA is very little.

However, the MondoA GKL139-141GAA and DEL139-141 mutants resulted in very low transcription activity for Txnip. The level of Txnip protein in GKL139-141GAA mutant group was significantly lower than wild type group (Figure 6D) and Txnip expression level showed almost the same as in the vector group, even when glucose was abundant in the medium (10 mM), and the nuclear entrance rate was not affected. Among these mutants, S287A had no significant effect on the expression of downstream Txnip, while mutants at positions 139-141, whether it is deletion mutation or non-sense mutation, will reduce the expression of Txnip. Therefore, it can be concluded that the 139-141 locus is a site where the small molecule G6P binds and activates MondoA, affecting the ability of MondoA to bind to the downstream gene promoter.

In summary, by some conventional experiments, we validated G6P is the mediate in glycolysis pathway which activates the transcription factor MondoA. Moreover, we evaluate the binding site with *in silico* methodologies, molecular docking and binding free energy calculation, and hMondoA139-141 residues are proved to be the binding site of G6P to activate MondoA (Figure S1). The result is confirmed both *in vitro* and *in vivo* by test-tube mutation approach, and eliminated the structure damage to the mutation. We believe this work is a significant step toward a better understanding of MondoA/Mlx-Txnip axis, and its connection with glucose metabolic flux.

## Discussion

MondoA undertakes a vital role in metabolism and multiple target genes are dedicated to its activity. The MondoA/Mlx-Txnip metabolic regulation pathway are implicated an substantial role in the occurrence and development of many diseases, such as the first and second most common causes of premature mortality, cancer (Abu el Maaty et al., 2017, 2019) and diabetes (Szpigel et al., 2018). Therefore, research on this pathway is an important aspect of identifying the cause and finding curable strategy. In diabetes and some non-solid tumors, such as blood cancers, the expression of MondoA is significantly increased (Wernicke et al., 2012; Sipol et al., 2017). If MondoA activity is inhibited, the expression of downstream Txnip and other MondoA-regulated genes will be significantly downregulated. Therefore, finding a site at which MondoA is activated by small molecules can serve as a target for inhibiting its activation so that damaged cells and reprogrammed cells can be rescued to minimum harmful states by altering the cellular metabolism.

It is still intricate to modulate its activity directly, even though the structure of MondoA has been studied thoroughly. Some inhibitors, such as SBI-477, are reported to be inhibitors of MondoA, and they can retard MondoA from entering the nucleus and may relieve insulin resistance in mice on a high-fat diet (Ahn et al., 2016). Additionally, there is reported that forskolin or the glucagon-like peptide 1 mimetic Exendin-4 (Richards et al., 2018) can inhibit the shuttle of MondoA in human pancreatic b-EndoC-bH1 cells, and almost all inhibitors which affect the activity of MondoA by adjusting its access to nucleus. While MondoA play the role as transcription factor in multiple proteins and it is cross talk in Max and Myc network (Wilde and Ayer, 2015; Qu et al., 2018). These interventions may involve the transmission of other signals, which in turn disrupts the precise and complex system of the cell. We believe that a better MondoA inhibitor should closely target the structural elements and activation mechanism of MondoA.

The exploration of the true activation site of MondoA ensures that the interference on cells is minimized. Moreover, the mutation we identified did not affect MondoA shuttles between OMM and nucleus. It proves that the different functionality between the mutant and the wild type stems from the three amino acid residues; the mutant MondoA cannot bind to the small molecule G6P, which affects the activation of MondoA. Therefore, even if it can enter into the nucleus, it will not occupy on the promoter to initiate the transcription of downstream genes. That is, the mutation of three amino acids in the MondoA MCR region changed the activation of MondoA by the small molecule metabolic intermediate G6P. If a small molecule that inhibits this region can be found, it could play an important role in the treatment of related diseases. We will conduct in-depth studies of this structure to develop better treatments for diseases caused by the overactivation of MondoA.

The molecule recognition approach provides us the brief and direct amino acids binding site of small molecules, which contributes grossly advantage to the study. We combined the conventional experiments with molecule docking and free energy decomposition analyses, and verified the activation of MondoA by the small molecule G6P and identified amino acid residues with which MondoA interacts with the small molecule G6P. The binding free energy calculation and decomposition is highly effective to compute the differences among mutations.

## Data Availability Statement

All datasets generated for this study are included in the article/Supplementary Material.

## Author Contributions

YL and XZ developed the study concept and design. XZ, TF, QH performed the experiments. XZ and TF carried out the data analysis. XZ drafted the manuscript, YL, XZ, and XG approved the manuscript.

### Conflict of Interest

The authors declare that the research was conducted in the absence of any commercial or financial relationships that could be construed as a potential conflict of interest.
