# Supplementary material for: Glucose-6-Phosphate Upregulates Txnip Expression by Interacting With MondoA
Source: Front Mol Biosci. 2020 Jan 9;6:147. doi: 10.3389/fmolb.2019.00147 (PMC6962712; doi:10.3389/fmolb.2019.00147)
Supplement: Supplementary file 1 [file Data_Sheet_1.pdf]

Glucose-6-phosphate upregulates Txnip expression by interacting with MondoA

Xue-Yun Zhang[1,2], Tao Fu[1,2], Qian He[1,2], Xiang Gao[1,2*] and Yan Luo[1,2*]

1. Department of Biochemistry and Cancer Institute of the Second Affiliated Hospital, Zhejiang University School of Medicine, Hangzhou, China;

2. Key Laboratory of Cancer Prevention and Intervention of China National Ministry of Education，Hangzhou，China

*Correspondence to Yan Luo: luoyan2011@zju.edu.cn or Xiang Gao: gaoxiang@u.nus.edu

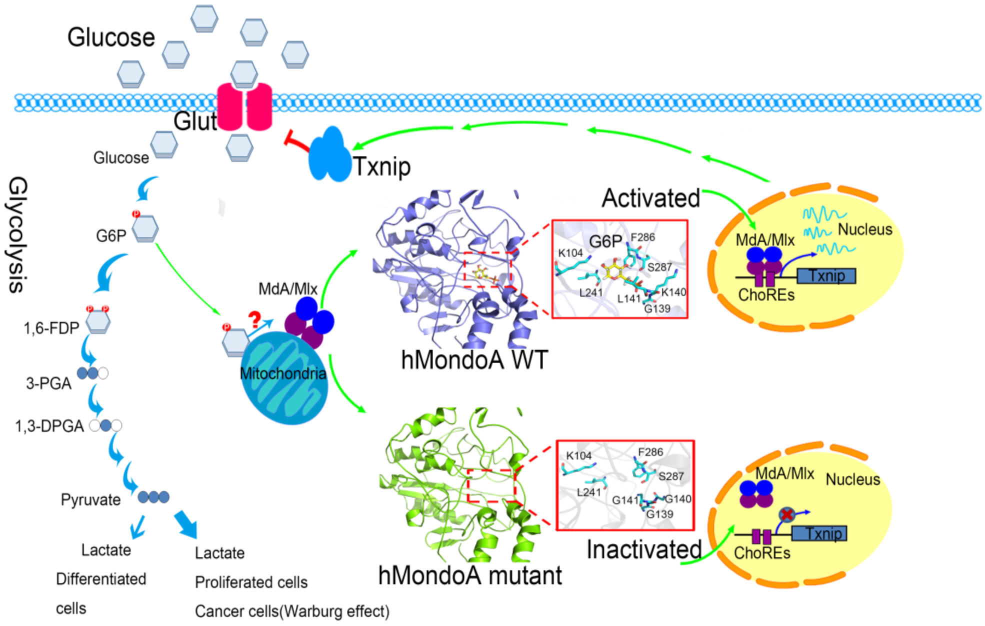

Figure S1 Schematic diagram for the expression of Txnip. G6P as an intermediate of glycolysis activated MondoA and induced the expression of Txnip by binding to hMondoA 139-141GKL.

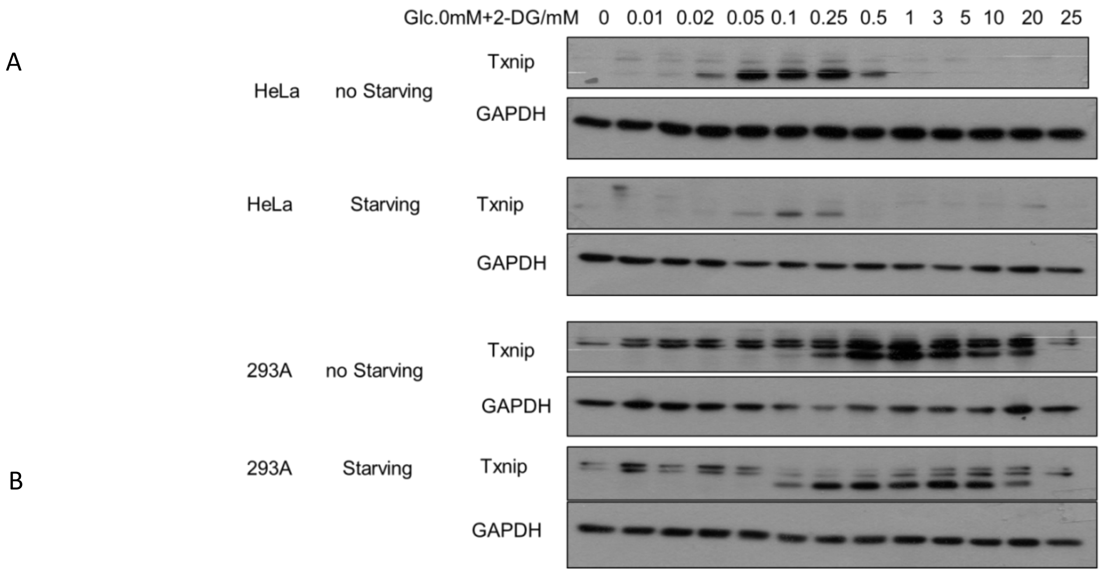

Figure S2 Changes in expression of Txnip after treating the cells with 2-DG 0 mM to 25 mM. (A)HeLa; (B) 293A.

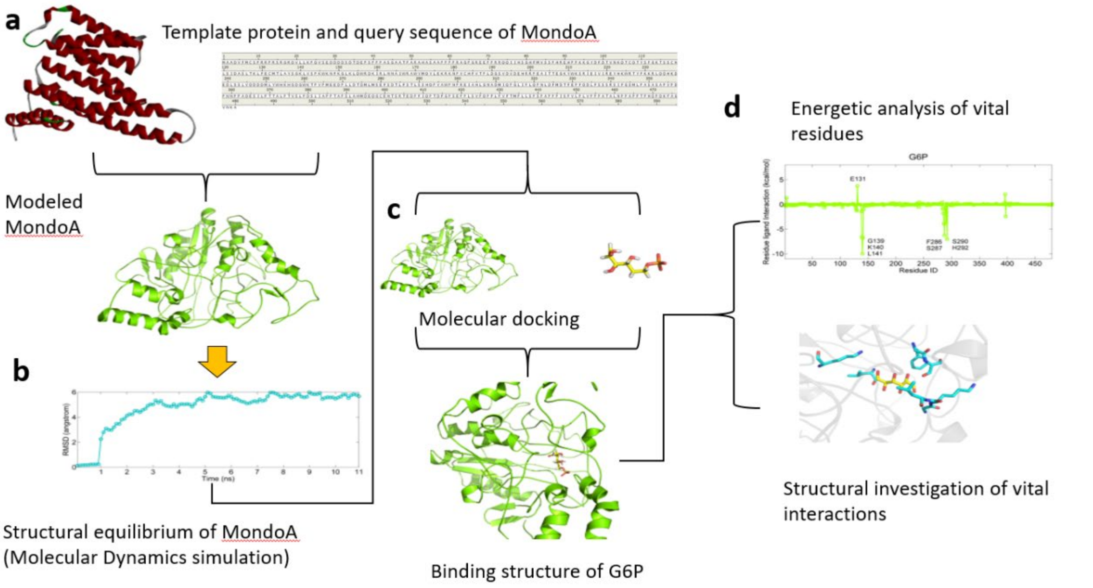

Figure S3 MondoA three-dimensional model modeling process demonstration. a) The Molecular Mechanics/Generalized Born Surface Area approach (MM/GBSA) was used for the binding free energy calculation and decomposition; b) Molecular Mechanics Minimization and Molecular Dynamics (MD) Simulation; c) Establishment of 3D model and flow chart of small molecule docking. MondoA three-dimensional model constructed by homology modeling; d) Deviation statistics (RMSD) for molecular dynamics optimization of the MondoA three-dimensional structural model after modeling.
